# Sesamin: A Promising Therapeutic Agent for Ameliorating Symptoms of Diabetes

**DOI:** 10.3390/molecules28217255

**Published:** 2023-10-25

**Authors:** Shu-Ming Huang, Cheng-Hung Chuang, Christine Joyce F. Rejano, Lemmuel L. Tayo, Cheng-Yang Hsieh, Steven Kuan-Hua Huang, Po-Wei Tsai

**Affiliations:** 1 Department of Clinical Nutrition, Nantou Hospital, Ministry of Health and Welfare, Nantou City 545, Taiwan; alice926112@gmail.com; 2 Department of Nutrition, Hungkuang University, Taichung City 433, Taiwan; chchuang@sunrise.hk.edu.tw; 3 School of Chemical, Biological, Materials Engineering and Sciences, Mapúa University, Manila 1002, Philippines; cjfrejano@mymail.mapua.edu.ph (C.J.F.R.); lltayo@mapua.edu.ph (L.L.T.); 4 School of Graduate Studies, Mapúa University, Manila 1002, Philippines; 5 Department of Biology, School of Medicine and Health Sciences, Mapúa University, Makati 1200, Philippines; 6 Ph.D. Program in Clinical Drug Development of Herbal Medicine, College of Pharmacy, Taipei Medical University, Taipei City 110, Taiwan; d339108001@tmu.edu.tw; 7 Laboratory of Oncology, Pharmacy Practice and Sciences, Graduate School of Pharmaceutical Sciences, Tohoku University, Sendai 980-8578, Japan; 8 Department of Medical Science Industries, College of Health Sciences, Chang Jung Christian University, Tainan 711, Taiwan; 9 Division of Urology, Department of Surgery, Chi Mei Medical Center, Tainan City 711, Taiwan; 10 School of Medicine, College of Medicine, Kaohsiung Medical University, Kaohsiung 807, Taiwan

**Keywords:** diabetes, *Sesamum indicum* L., sesamin, insulin resistance, streptozotocin

## Abstract

Diabetes is a chronic metabolic disease characterized by improperly regulating proteins, carbohydrates, and lipids due to insulin deficiency or resistance. The increasing prevalence of diabetes poses a tremendous socioeconomic burden worldwide, resulting in the rise of many studies on Chinese herbal medicines to discover the most effective cure for diabetes. Sesame seeds are among these Chinese herbal medicines that were found to contain various pharmacological activities, including antioxidant and anti-inflammatory properties, lowering cholesterol, improving liver function, blood pressure and sugar lowering, regulating lipid synthesis, and anticancer activities. These medicinal benefits are attributed to sesamin, which is the main lignan found in sesame seeds and oil. In this study, Wistar rat models were induced with type 2 diabetes using streptozotocin (STZ) and nicotinamide, and the effect of sesamin on the changes in body weight, blood sugar level, glycosylated hemoglobin (HbA1c), insulin levels, and the states of the pancreas and liver of the rats were evaluated. The results indicate a reduced blood glucose level, HbA1c, TG, and ALT and AST enzymes after sesamin treatment, while increased insulin level, SOD, CAT, and GPx activities were also observed. These findings prove sesamin’s efficacy in ameliorating the symptoms of diabetes through its potent pharmacological activities.

## 1. Introduction

Diabetes is among the most prevalent chronic diseases and is characterized by improper regulation of proteins, carbohydrates, and lipids due to insulin deficiency or insulin resistance [1]. Indications of diabetes are shown to be contingent on the gravity of the metabolic malfunctions ranging from reduced body weight to increased blood glucose level and even comatose. The different symptoms of diabetes resulted in the classification of this disease into four types. Malfunctions in the autoimmune system cause damage to insulin-producing beta cells and insulin resistance due to failing insulin receptors, which are characterized as type 1 and type 2 diabetes, respectively. The remaining types of diabetes (gestational and prediabetes) are both related to type 2 diabetes. Often inflicted by gestational diabetes are women that are pregnant [2]. Diabetes can further impose illnesses such as cardiovascular disease, chronic renal failure, retinopathy, neuropathy, and microvascular disease. Microvascular disease may result in dysfunctional sexual function and impediments to wound healing. Non-healing foot wounds can cause gangrene, resulting in amputation. Due to various health risks associated with diabetes, finding a permanent cure is needed. Gestational diabetes usually resolves on its own after pregnancy. Although conventional therapies for managing type 1 and 2 diabetes are available, finding a definite cure for type 1 and 2 diabetes remains challenging. Diabetes is pervasive, and it caused significant damaging effects on society. Today, the estimated number of people that are imposed with diabetes mellitus is around 422 million people, and it is accountable for the death of 1.5 million people a year worldwide [1].

The increasing prevalence of diabetes poses a tremendous socioeconomic burden worldwide, more specifically to Asian communities. According to a study, from the year 2011 to 2030, the forecast for the number of diabetes would be accounted from 366 million people to 552 million, respectively, with half coming from Asian countries based on the International Diabetes Federation (IDF) [3]. Recently, the IDF also issued data demonstrating that the highest countries with diabetes spreading rapidly are China, India, and Pakistan [4]. Today, there is a great presumption that the current number of 140.9 million individuals in China inflicted with diabetes will continue to increase by 33.5 million people by 2045, totaling 174.4 million individuals [5]. Therefore, several Chinese herbal medicine studies are being conducted in an effort to find the most effective medication that can lower blood sugar as diabetes continue to prevail and affect not just people, but also society and its economy. Among the Chinese herbal medicines are sesame seeds. Sesame is the dry seed of *Sesamum indium* L., also known as flax or linseed.

Sesame is among the ancient oilseed crops and China’s four major oil crops used to prepare a variety of food and garnish other cuisines. Various studies concentrated on sesame as its primary subject, some of which already tested its fundamental functional compounds. As such, results were weighed out, and some of the compounds shown were the sesamin, sesamolin, sesamol, sesaminol, phenol, and other lignan-like active ingredients, which all play a crucial role in curing and alleviating potential diminishing chronic diseases [6,7,8,9]. Based on studies, in the year 2002, the former Chinese Ministry of Health formally introduced and recognized sesame seeds as having medicinal and dietary value for herbal medications and treatments [10]. Previous research on the processing and consumption of sesame focuses mainly on studying its nutritional function and composition, which includes vitamins, minerals, protein composition, and amino acid profile. Sesame seed contains great amounts of vitamin B1, dietary fiber, proteins, and important minerals such as iron, calcium, magnesium, copper, zinc, and phosphorous [11]. In addition to these valuable components, sesame is also a good source of vitamin E. Vitamin E is a phenylpropanoid derivative, and its chemical structure has -OH group, which has a reducing action and can also provide electrons [12]. The five-ring structure also has a saturated hydroxyl chain, indicating that vitamin E possesses both reducing and lipophilic properties [13]. Literature also indicates that sesame seeds contain 50–60% high-quality oil that is rich in bioactive components such as polyunsaturated fatty acids, lignans, tocopherols, and phytosterols [11,14]. These active ingredients were found to provide a wide range of health benefits, including anti-lipogenic, anti-atherogenic, anti-inflammatory, antioxidant, anti-cancer, cholesterol-lowering, anti-degenerative, and cardio-protective effects [11,14,15,16,17,18,19]. As the research goes deep into analyzing the composition of chemical components to explore active substances, in recent years, the focus of research shifted to the function of sesame lignans. Sesame lignans (sesamin, sesamolin, sesaminol, sesamolinol, and sesamol) are the primary active components of sesame seeds, which are reported to contribute to sesame’s health-promoting effects. The major lignans in sesame seeds are sesamin and sesamolin, which were found to constitute 0.1–0.5% of sesame. These lignans were reported to exhibit antioxidant and anticancer activities, neuroprotective properties, cholesterol-reducing effects, and anti-osteoarthritis [16,17,18,19,20,21,22,23]. Other derivatives of sesame (i.e., sesaminol, sesamolinol, and sesamol) were also found to possess pharmacological properties (e.g., anti-inflammatory, antioxidant, anti-aging, and anti-hepatotoxic activities), albeit small amounts [24,25,26].

In addition, prior studies on sesame revealed that sesame extracts also have the potential to ameliorate symptoms of diabetes in STZ-induced diabetic mice [27,28,29,30]. A study conducted in 2016 demonstrated that sesamin treatment resulted in a drop in blood glucose levels, suppression of microglial activation, and reduction in levels of retinal TNF-α and ICAM-1 [29]. These effects were observed to impede the progression of diabetic retinal injury. Sesamin was also shown to lower fasting blood glucose, cholesterol levels, and triglyceride levels, which improves diabetes conditions in diabetic mice [27]. Consistent with this finding, a comparable investigation also documented the capacity of sesame extract to mitigate hyperglycemia and ameliorate nephropathy in STZ-induced mice [30]. Streptozotocin (STZ) is described as a cell-killer toxin that targets pancreatic β-cells, mainly destroying pancreatic islet cell carcinoma that cannot be removed by surgery [31]. STZ is also an agent commonly used in inducing diabetes in animal models. Nicotinamide, on the other hand, is a derivative of vitamin B3, which can be used to counteract the effects of STZ by reducing the toxicity induced by STZ and protecting pancreatic β cells from being completely destroyed [31]. The pharmacological studies of compounds present in *Sesamum indicum* (Linn) seeds in the management of diabetes are scarce. Thus, this research aims to evaluate the efficacy of sesamin (the most abundant compound in sesame seeds) in alleviating the symptoms of diabetes (i.e., body weight loss, high blood glucose level, and glycosylated hemoglobin, low insulin level, decrease in serum amylase activity accompanied by a decrease in high-density lipoprotein (HDL), elevated serum triglyceride (TG), increased cholesterol, lowered serum amylase, and hepatic injuries) using STZ-induced rat models.

## 2. Results

In this study, type 2 diabetes was induced in Wistar rats as test models. The effects of sesamin treatment on the STZ-induced diabetic rats were evaluated by observing the changes in their body weight, blood sugar level, glycosylated hemoglobin, insulin level, number of pancreatic islet cells, and biological serums.

### 2.1. The Effect of Sesamin on the Body Weight of STZ-Induced Type 2 Diabetic Rats

**Figure 1:**
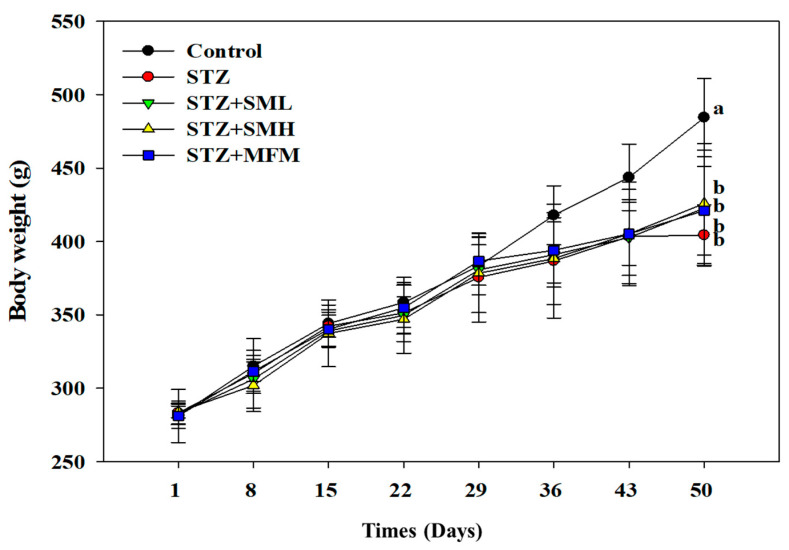
Effects of sesamin on the body weight of STZ-induced diabetic rats. The data (mean ± standard deviation) were collected from three or four independent experiments, and the means for each cell type measured at the same incubation time exhibit statistically significant differences (*p* < 0.05) when they do not share a common alphabetic letter (a, b).

The results are presented in Figure 1. The changes in the body weights of both control and diabetic rats varied as the experiment progressed. There was no observed significant difference (*p* > 0.05) between the control group and the STZ-induced diabetic rats from day 0 to 35. However, on the 36th day of the experiment, a 7.4% significant difference was recorded, while a 9.1% difference was observed on the 43rd day. Furthermore, the body weights of the diabetic rats continued to decrease until the 50th day by 16.5%. The rest of the groups showed no significant difference from the STZ treatment group (*p* > 0.05). As observed in this experiment, the rats treated with STZ experienced weight loss. However, diabetic rats that were treated with metformin (MFM) and high and low dosages of sesamin seemed to manage the weight-reducing effects of STZ in diabetic rats. As shown in Figure 1, STZ-induced rats that were not treated with any medication experienced the greatest amount of weight loss compared to those treated with sesamin and metformin. This effect may indicate sesamin’s potential in alleviating significant body weight loss due to diabetes.

### 2.2. The Effect of Sesamin on the Blood Sugar Level in STZ-Induced Type 2 Diabetic Rats

**Figure 2:**
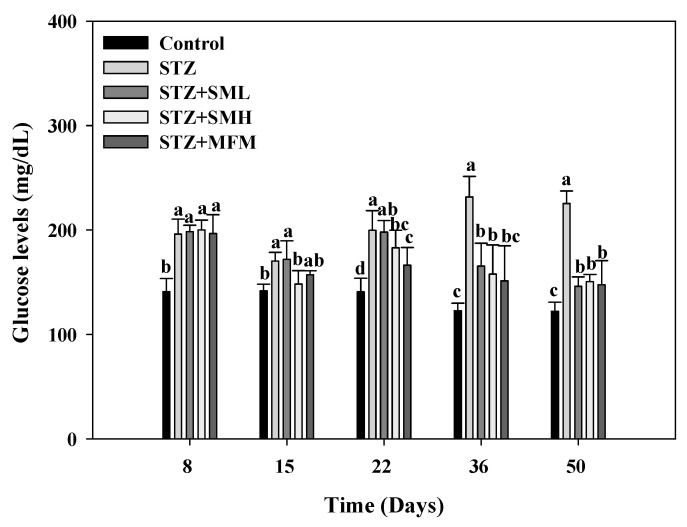
The effect of sesamin on the blood sugar of STZ-induced type 2 diabetes in rats. The data (mean ± standard deviation) were collected from three or four independent experiments, and the means for each cell type measured at the same incubation time exhibit statistically significant differences (*p* < 0.05) when they do not share a common alphabetic letter (a, b, c, d).

In order to determine the blood glucose level of the treatment group, streptozotocin or STZ was administered on the 8th day of the experiment and was compared to the control group. The group given STZ had higher levels compared to the control group in Figure 2 (190 mg/dL, *p* < 0.05). By days 15 and 22, the results show that the STZ group still had notable differences compared to the control group. The results of day 8 and day 15 from the STZ group, however, show that the glucose level on day 15 was lower than on day 8 by 13.3% (*p* < 0.05). As such, the researchers decided to readminister STZ to each group on day 15, and it was found that glucose levels on day 22 increased by 219% (*p* < 0.05). STZ was then readministered every two weeks to control the glucose levels of the STZ group. Successful administration of STZ to induce diabetes in the rats was confirmed by the sudden increase in blood glucose levels of the rat models. It can also be observed in Figure 2 that the STZ group exhibited a continuous increase in blood glucose levels, but the groups that received sesamin and metformin treatments showed a gradual decrease in glucose levels. The comparable effects of metformin and sesamin in lowering the blood glucose levels of diabetic rats observed in this experiment imply the potential of sesamin in diabetes treatments.

### 2.3. The Effect of Sesamin on the Glycosylated Hemoglobin in STZ-Induced Type 2 Diabetic Rats

**Figure 3:**
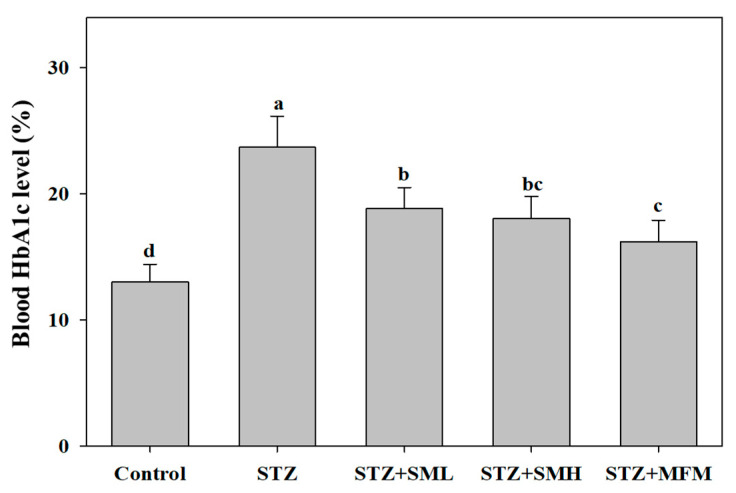
Effect of sesamin on glycosylated hemoglobin in STZ-induced type 2 diabetes rats. The blood samples were collected from the retro-orbital plexus of the rats under deep isoflurane anesthesia to determine HBA1c levels on day 50. Values (means ± SD, *n* = 3) not sharing a common lower case letter are significantly different (*p* < 0.05).

On the eighth day of the experiment, the following drugs were given: SML, SMH, and MFM. Results demonstrate that when comparing the STZ control group with that of the SML-treated group, HbA1c levels show result deeming that the difference between the two was not significant on the 15th to 22nd day with a *p*-value being greater than 0.05 but with a notable difference on the 36th to 50th day of the experiment with *p*-value less than 0.05, resulting in a decrease of 28.6% and 35.2%, correspondingly (Figure 3). Compared to the STZ group, SMH showed a drop (*p* < 0.05) in HbA1c by 12.8%, 11.7%, 28.4%, and 33.2%, respectively. On the other hand, the MFM group dropped (*p* < 0.05) 7.5%, 19.7%, 34.75, and 34.5%, respectively, compared to the STZ group. The results clearly show that sesamin and metformin can reduce the HbA1c levels in STZ-induced diabetic rats, which may imply the positive effects of the treatments.

### 2.4. The Effect of Sesamin on Insulin Levels in STZ-Induced Type 2 Diabetic Rats

**Figure 4:**
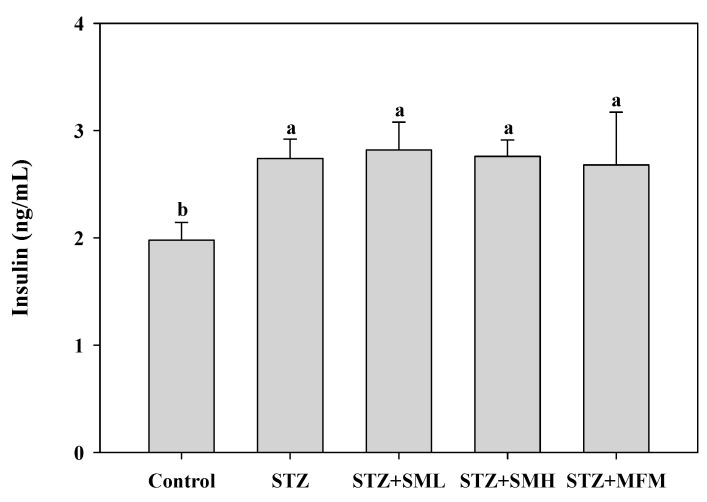
Effect of sesamin on insulin in STZ-induced type 2 diabetes in rats. The blood samples were collected from the retro-orbital plexus of the rats under deep isoflurane anesthesia to determine serum insulin levels on day 50. Values (means ± SD, *n* = 3) not sharing a common lower case letter are significantly different (*p* < 0.05).

In comparing the two groups, control and STZ treated, results display a 38% significantly greater insulin intensity for the STZ-treated group, with a *p*-value resulting to less than 0.05 (Figure 4). On the other hand, no significant differences were discovered between the other groups and the STZ-treated group (*p* > 0.05). As illustrated in Figure 4, the injection of sesamin in rats with STZ-induced type 2 diabetes led to a notable enhancement in insulin sensitivity as compared to the control group. It is important to take note that enhanced insulin secretion or improved insulin sensitivity is crucial in managing diabetes. Although the difference between the STZ-treated group and the other treatment groups is not statistically significant, it does not necessarily mean that the treatment conducted is ineffective. In this case, increasing the sample size and the refinement of experimental parameters can be the future direction of this study in order to enhance the statistical significance of the results.

### 2.5. The Effect of Sesamin on the Pancreas of STZ-Induced Type 2 Diabetic Rats

**Figure 5:**
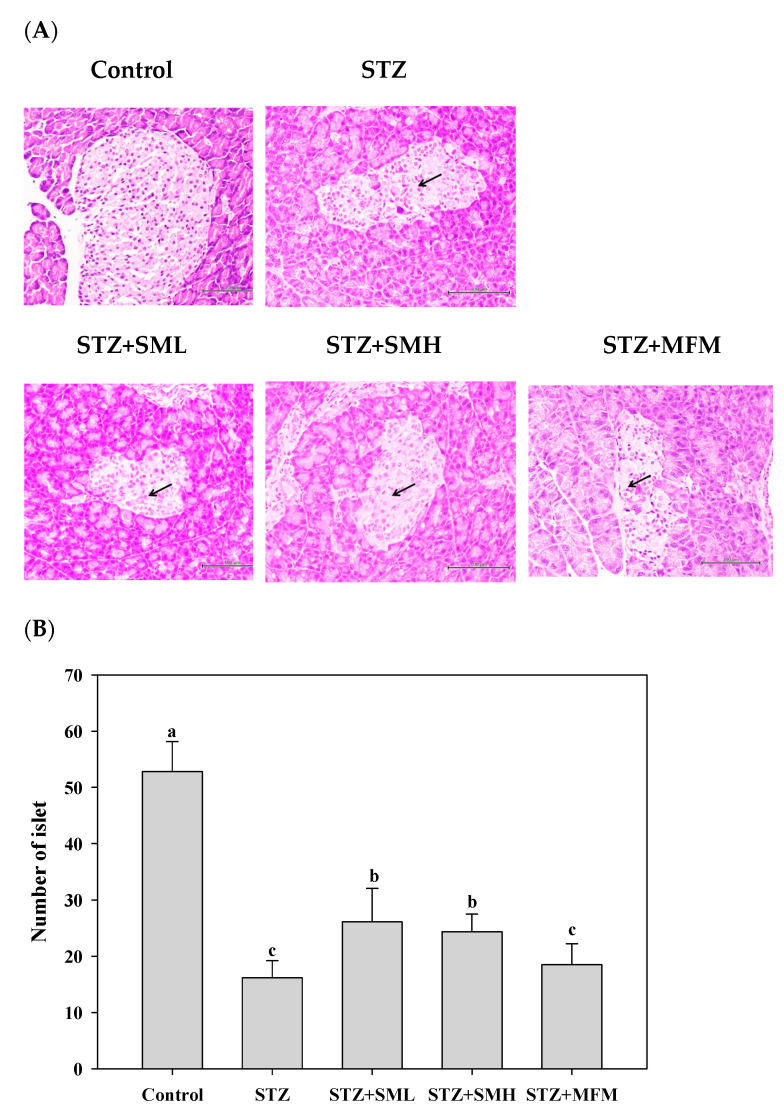
The effect of sesamin on the pancreas of STZ-induced type 2 diabetes in rats: (**A**) histopathology result of pancreatic tissues; (**B**) number of islet cells in the pancreas. The data (mean ± standard deviation) were collected from three or four independent experiments, and the means for each cell type measured at the same incubation time exhibit statistically significant differences (*p* < 0.05) when they do not share a common alphabetic letter (a, b, c). The black arrow indicates that normal β cells are located in the central area with abundant cytoplasm, while β cells with less cytoplasm surround β cells in the peripheral area of the islet in a control pancreas. Islets in the pancreas after treatment with STZ in rats showed necrosis with mononuclear cell infiltration, as well as significant atrophy and a decrease in β cells.

The findings presented in Figure 5A depict the outcomes obtained from the investigation of islet cells inside pancreatic tissues, particularly on β cells. The present investigation assessed the variations between the control and STZ-induced diabetic groups, revealing a decrease in β cell apoptotic cells and an increase in these cells, respectively. These results are indications that there is no significant development in the β-cell apoptosis symptoms carried by the STZ. However, the number of islet cells in the pancreas was considerably decreased by 242% in the STZ treatment group as compared to that of the control group (Figure 5B). The low and high doses of SM in each treatment group and the MFM treatment group can significantly augment the reduction in islet cells caused by STZ, reaching 70%, 58%, and 20%, respectively. These findings indicate that sesamin, particularly at elevated dosages, and metformin could exhibit protective properties towards the insulin-producing islet cells located in the pancreas, which play a crucial role in the regulation of blood glucose levels.

### 2.6. The Effect of Sesamin on Serum Biochemical Values of STZ-Induced Type 2 Diabetic Rats

Table 1 demonstrates the effects of sesamin treatment on the values of different biochemical serums. The study found that between the control and STZ-treated group, the amounts of alanine aminotransferase (ALT), aspartate aminotransferase (AST), and triglycerides (TG) serums are significantly greater for the control group than that of the treated group by 110%, 54%, and 37%, correspondingly (*p* < 0.05). In line with this, the study also found that the concentrations of ALT, AST, and TG in serum are possibly reduced by the treatment groups: SML, SMH, and MFM. Increased levels of ALT and AST in the blood may serve as indicators of hepatic injury or inflammation. Elevated levels of TG are often associated with insulin resistance and metabolic disturbances in diabetes. In this study, varying inhibition rates were obtained upon administration of sesamin and metformin treatments. ALT inhibition rates of 19%, 30%, and 30% and AST inhibition rates of 15%, 24%, and 43% were obtained from groups treated with SML, SMH, and MFM, respectively, suggesting a potential reduction in liver injury induced by STZ. Moreover, TG inhibition rates were 23%, 52%, and 34%. This finding implies that the administration of sesamin, particularly in high dosages, may potentially aid in lowering increased triglyceride levels, hence offering advantages in terms of metabolic regulation and cardiovascular health. Furthermore, no difference was observed between the total cholesterol (TC) results of the STZ group and the control group (*p* > 0.05). The SM groups of both high and low doses and the STZ group also showed no real change (*p* > 0.05). However, there was a 14% increase observed in the MFM group compared to the STZ group (*p* < 0.05). In addition, the results for the high-density lipoprotein (HDL), when the STZ group was treated with the serum HDL concentration, are lower by 18%. Including the high-dose group of sesamin (SMH) and the MFM group, each treatment group showed an improved decrease, by 21% and 18%, respectively, in serum HDL concentration from STZ. The low-dose group of sesamin (SML) did not show a significant decrease in the serum HDL concentration from STZ (*p* > 0.05). Results further show that when the control and STZ treatment groups were differentiated, STZ-treated groups had a lower concentration of amylase compared to the control group by 24%. The reduction in serum amylase concentration that is specifically induced by the STZ (*p* < 0.05) can be halted through the administration of the following treatment groups: SML, SMH, and MFM treatment with their respective percentages of 38%, 49%, and 27%.

These results suggest that administering sesamin, especially at higher dosages, may have a positive effect on many serum biochemical markers, mitigating the effects caused by STZ in the rat model used for experimentation.

### 2.7. The Effect of Sesamin on the Liver of STZ-Induced Type 2 Diabetic Rats

**Figure 6:**
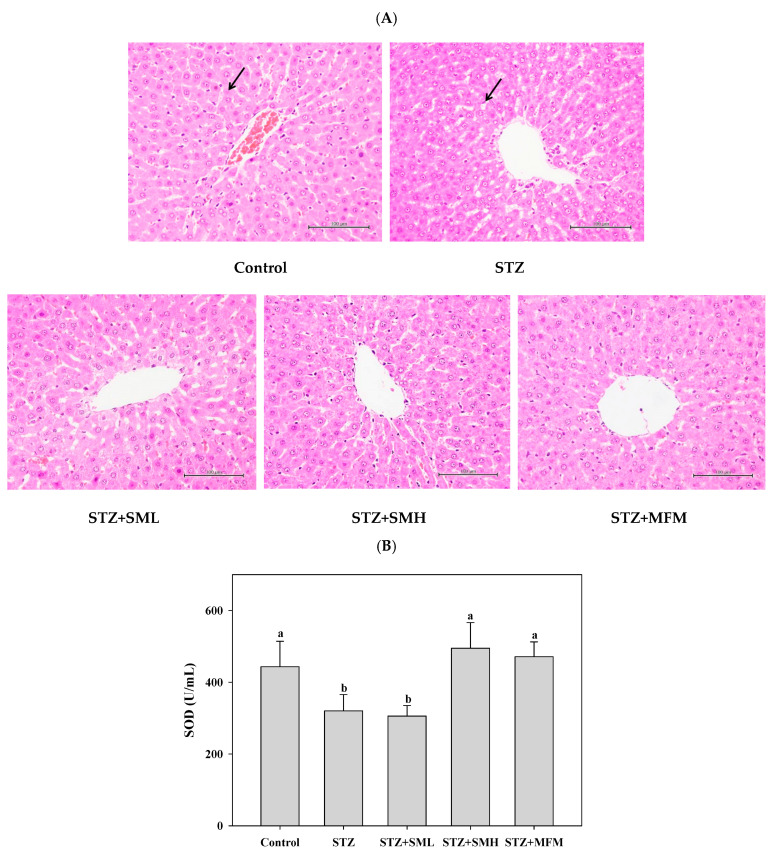

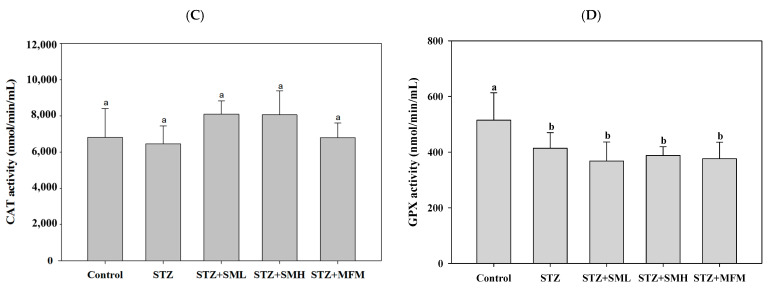
Effects of sesamin on the liver of STZ-induced type 2 diabetes in rats: (**A**) histopathology results of the liver tissue sample of diabetes-induced rats; (**B**) SOD concentration in liver tissue; (**C**) CAT activity in the liver; and (**D**) GPx activity. The data (mean ± standard deviation) were collected from three or four independent experiments, and the means for each cell type measured at the same incubation time exhibit statistically significant differences (*p* < 0.05) when they do not share a common alphabetic letter (a, b). The black arrow indicates the nuclear gap in the liver tissue of the STZ-induced diabetic group, which is closer than that in the normal group.

The liver tissues of the STZ-induced diabetic rats were observed throughout the experiment in order to determine the effect of sesamin treatment. The results are shown in Figure 6A–D. Figure 6A demonstrates that all groups exhibit no significant lesions in their liver tissues. When comparing the control group, outcomes of antioxidant enzymes (Figure 6B–D) displayed that the SOD concentration in liver tissues showed a greater reduction of 28% for the STZ treatment group than that of the control group. Results also show that the deduction of concentration in SOD being caused by STZ between the two treatment groups, SM high-dose and MFM, can majorly develop both by reaching 55% and 47%, respectively. The SML treatment group did not significantly improve the reduction in SOD in liver tissue caused by STZ (*p* > 0.05; Figure 6B). However, results also display that among the liver tissues of the groups, no significant difference between the concentration of CAT was shown (*p* > 0.05; Figure 6C). Results also demonstrate that there was a significant reduction of about 20% GPx concentration in liver tissues of the STZ treatment group as compared to that of the control group with a *p*-value less than 0.05. Furthermore, the difference between the other groups and the STZ treatment group showed no significance (*p* > 0.05; Figure 6D). With these, the results observed in this experiment may indicate that the administration of sesamin, especially at higher dosages, may exhibit a beneficial impact on the levels of antioxidant enzymes in the liver tissues of rats with diabetes caused by STZ. Moreover, sesamin treatment appears to counteract the decrease in SOD and GPx concentrations caused by STZ, suggesting a possible involvement in reducing hepatic oxidative stress.

## 3. Discussion

In preceding studies that focused on sesame, sesamin was found to possess substantial pharmacological activities that make it a potential candidate for alleviating diabetes symptoms through antioxidant and anti-inflammatory activities [32,33,34,35]. In this study, the positive effects of sesamin in diabetic rats are elucidated and are consistent with related literature. The results of this experiment reveal that rats treated with STZ experienced decreased body weight, whereas the control groups maintained their normal body weight, and this difference was statistically significant. This effect can be correlated with the degradation of structural proteins since structural proteins significantly contribute to body weight [36]. According to the findings of Ramesh et al. (2005), the ability of sesame oil to control hyperglycemia may have a direct correlation with its capacity to prevent maximum weight loss [37]. Given that sesamin make up almost half of the sesame component [8], it could also be the possible reason why diabetic rats treated with sesamin (SML and SMH) did not reduce much weight.

Furthermore, the present study observed a significant reduction in blood glucose levels and glycosylated hemoglobin (HbA1c) in diabetic rats treated with sesamin, as compared to the control group of diabetic rats. Based on studies, the significant reduction in blood glucose levels and hemoglobin can be attributed to sesamin’s ability to stimulate glycogen production in the liver [27,37,38]. Lignans such as sesamin found in sesame seeds were reportedly capable of regulating gene expression associated with insulin signaling, glycogen synthesis, and glucose metabolism in type 2 diabetic rats [39]. Moreover, Hong et al. suggested that sesamin plays a role in increasing glycogen synthesis, which prevents a significant increase in blood glucose [27]. According to Lei et al., increased insulin levels and reduced blood glucose levels due to sesamin can be attributed to its protective impact on the damaged NIT-1 pancreatic β-cells produced by STZ, which may be related to its ability to lower oxidative stress and NO production (β-cell-destructive agents) [40]. Streptozotocin (STZ) is particularly toxic to the insulin-producing beta cells of the pancreas in mammals. As a result of the streptozotocin action, beta cells undergo the destruction by necrosis [41]. However, in this study, there was no significant decrease in insulin levels in the group treated with STZ alone. Therefore, it is suggested that beta cells are resilient and will compensate to cope with insulin demand despite reduced numbers [42], but the mechanism still needs to be further studied.

Consequently, after the administration of sesamin in diabetic rats, the results show a considerable increase in the antioxidant capacity of the liver, suggesting a protective effect against oxidative stress. Figure 6 demonstrates that SOD, CAT, and GPx significantly decreased in STZ-induced diabetic rats. These results are consistent with other research findings [43,44,45,46]. Sesamin, however, enhanced the oxidative status of diabetic rats by boosting the levels of SOD and CAT. Increased SOD activity indicates that sesamin may enhance the capacity to remove superoxide produced during oxidative stress [47]. At the same time, increased CAT activity in diabetes is indicative of higher peroxide radical generation to overcome oxidative stress [48]. Glutathione peroxidase (GPx) was also expected to increase in sesamin-treated groups since it is an antioxidant enzyme essential for protecting cells from the damaging effects of hydrogen peroxide [27,46,49].

Lipids were also reported to have a crucial role in the development and manifestation of diabetes. Regardless of insulin resistance or insulin insufficiency, abnormalities in serum lipid levels are frequently observed in diabetic populations [50]. The insufficient insulin in the body leads to an elevated blood glucose level, along with elevated serum triglyceride (TG), increased cholesterol, lowered serum amylase, and a decrease in high-density lipoprotein [51]. In line with previous studies, the cause of hyperglycemia in animals was due to the interaction between the growth and expansion of triglyceride generated by the deficit of insulin levels [52]. Previous studies also found that diabetic patients experience a decrease in serum amylase activity accompanied by a decrease in HDL, suggesting a direct relationship between the two biological elements [53]. Impaired insulin is attributed to low serum amylase levels because insulin resistance is being secreted, as observed in the glucose levels of diabetic individuals [53]. However, the administration of sesamin significantly improved the lipid profile of diabetic rats. In this experiment, the diabetic rats treated with sesamin showed a significant decrease in TG and an increase in HDL and amylase. This result also agrees with other research findings [54,55]. According to Tai et al., sesamin may enhance blood lipid levels by lowering hepatic steatosis and intestinal cholesterol absorption [56]. In addition, sesamin was also found to have a lipid metabolizing effect by inhibiting major steps in fatty acid synthesis [57]. This may indicate the sesamin’s ability to decrease the adverse effects of diabetes.

Lastly, a few indicators that may also suggest that an individual is inflicted with diabetes are the excessive levels of aminotransaminase or AST and serum alanine aminotransaminase or ALT, which may also be related to hepatic injury [58,59]. The increase in the activities of AST and ALT in STZ-induced diabetic rats in this study suggests the detrimental effect of STZ on the liver, which caused hepatic cells to leak from cytosol to the bloodstream [60]. Sesamin and metformin treatment for diabetic rats were shown to considerably reduce the levels of these enzymes, which may indicate a reversal of the adverse effects of elevated blood glucose in diabetic rats and, as a result, ameliorate liver damage caused by STZ [54].

## 4. Materials and Methods

This experiment was performed according to the experimental design presented in Table 2. A total of 30 adult male Wistar rats were used during the experiment. The rats were divided into five groups: (1) control group, (2) STZ-treated group, (3) SML-treated group, (4) SMH-treated group, and (5) MFM-treated group as the positive control. The doses of sample given to each treatment group were tabulated, as shown in Table 2.

### 4.1. Animal Experiment and the Effect of Sesamin on the Body Weight

Adult male Wistar rats aged six weeks were purchased from BioLASCO Taiwan Co., Ltd., Taipei, Taiwan. Afterwards, on days 1, 15, 29, and 43 of the experiment, rat groups 2 to 5 were given 200 mg/kg/body weight (dissolved in PBS, pH 7.4) of nicotine through intraperitoneal injection after they acclimatized for a week duration. Together with this, after a period of 15 min, STZ with an amount of 55 mg/kg/body weight (dissolved in 0.1 M citrate buffer, adjusted to pH = 4.5) was intraperitoneally injected in order to induce diabetes into the rats. Meanwhile, through the use of a tube, rat groups 3 to 4 were fed with sesamin each day with an amount of about 20 and 60 mg/kg/body weight (dissolved in 5% carboxymethylcellulose) of sesamin, which started on the 8th day of the experiment. Comparably, 250 mg/kg/body weight (dissolved in 5% carboxymethylcellulose) metformin was fed into group 5 every day to serve as the positive control group. For 50 days of the experiment, the blood glucose level was monitored through accumulating blood samples. Sacrifice was after the end of the experiment on the 50th day, and the blood and organs were removed for analysis. The experimental protocol was approved by the Ethics Committee of Hungkuang University (Approved no. HK-10906), and significant measures were taken to minimize animal distress.

### 4.2. Blood Glucose Monitoring

The rats were subjected to 8 h fasting prior to the collection of blood samples. After eight hours, blood was accumulated from the tail vein, and the blood sugar level was assessed by utilizing the one-touch blood glucose machine.

### 4.3. Detection of Glycosylated Hemoglobin (HbA1c)

Prior to the sacrifice, the blood collection was conducted eight hours after abstaining from food through the eye socket blood collection. Meanwhile, using the blood tubes that held K2EDTA, blood was collected, and the tools HbA1c HPLC Assay Kit (Catalog Number: A1C31-H100, Eagle Biosciences, Amherst, NH, USA) were utilized in order to analyze HbA1c levels.

### 4.4. Determination of Serum Insulin Concentration

An ELISA kit was used to quantify the insulin concentration in rats. A total of 25 μL of calibrator and serum was placed into a 96-well plate and washed with wash buffer six times. Then, the TMB reagent was added. After the TMB reagent was reacted for 15 min, 50 μL of stop solution was added to the mixture. Afterward, an enzyme immunoassay reader (ELISA Reader (Cat No: BioTek EPOCH2, Agilent Technologies, Inc., Santa Clara, CA, USA)) was used to measure the absorbance at a wavelength of 450 nm.

### 4.5. Determination of Plasma Triglycerides, Cholesterol, Amylase, HDL, ALT, and AST

A biochemical blood analyzer (FUJI-DRC-CHEM 3500, Fujifilm, Kanagawa, Japan) and commercially available test paper were used to determine the biochemical content of the samples. Subsequently, 10 μL was dispensed onto the test paper and then placed inside the test paper tank. Then, the paper was read using a blood biochemical analyzer to determine the biochemical value immediately.

### 4.6. Antioxidant Enzyme Assay

The liver tissue was obtained after the sacrifice of the rat for antioxidant enzyme assay.

#### 4.6.1. Determination of Glutathione Peroxidase (Glutathione Peroxidase, GPx)

In establishing the GPx concentration in liver tissues, the commercial GPx assay kit (Cayman, Ann Arbor, MI, USA, Item No. 703102) was utilized. Next, 0.1 g of liver tissue were taken and added into a homogenized tube, and 1 mL of homogenization solution (50 mM Tris-HCl, 5 mM EDTA, and 1 mM DTT) were mixed in a tube and next placed in ice. Afterward, tissues were grounded using the tissue grinder (Glas-Col 099D GT31, Glas-Col, Terre Haute, IN, USA) and then centrifuged at 10,000 rpm at 4 °C for 15 min. Subsequently, the reagents and supernatant were mixed and incubated at room temperature for ten minutes to make them react. Once reacted, the absorbance at wavelength 340 nm was measured using an enzyme immunoassay reader (ELISA Reader).

#### 4.6.2. Determination of Superoxide Dismutase (SOD)

The commercial GPx assay kit (Cayman, Ann Arbor, MI, USA, Item No. 706002) was utilized in order to establish the GPx concentration in liver tissues; 0.1 g of liver tissue was placed inside a tube. Then, 1 mL of homogenization solution (20 mM HEPES buffer, 1 mM EGTA, 210 mM mannitol, and 70 mM sucrose) was added into the same tube and placed in ice. Afterward, tissues were grounded by utilizing the tissue grinder (Glas-Col 099D GT31) and were centrifuged at 1500 rpm at 4 °C for 15 min. Subsequently, the reagents and supernatant were mixed and incubated at room temperature for ten minutes to make them react. Once reacted, the absorbance at wavelength 450 nm was measured using an enzyme immunoassay reader (ELISA Reader).

#### 4.6.3. Determination of Catalase (CAT) Activity

The concentration of CAT in liver tissue was determined using a commercial SOD assay kit (Cayman Ann Arbor, MI, USA, Item No. 707002), and 0.1 g of liver tissue were taken and added into a homogenized tube, and 1 mL of homogenization solution50 mM potassium phosphate and mM EDTA were mixed in a tube and were next placed in ice. Afterward, a tissue grinder (Glas-Col 099D GT31) was used to grind the tissue. The samples were centrifuged (10,000 rpm, 4 °C, 15 min). The supernatant was mixed evenly with the reagents and placed at room temperature for 10 min until it reacted. Once reacted, the absorbance value at wavelength 450 nm was measured using an enzyme immunoassay reader (ELISA Reader).

### 4.7. Histological Studies

#### 4.7.1. Tissue Embedding and Sectioning

The tissue pieces of about 0.5 cm squares that were removed from the pancreas were placed in 10% neutral formalin to fix their tissue shape and structure. Then, ethanol with different concentrations (30%, 50%, 70%, 95%, and 99.5%) and xylene were used for the dehydration and clearing steps, respectively. After the clearing steps, xylene was replaced with hot paraffin solution and finally embedded the tissue with paraffin solution. The completed paraffin specimens were cut into 5 μm serial paraffin sections with a microtome, and the cut sections were stuck on clean glass slides, dried at 37 °C, and used for further pathological staining.

#### 4.7.2. Haematoxylin-Eosin (H&E) Staining

Tissues of the pancreas were soaked for 30 min in xylene to be deparaffinized. After the deparaffinization, the tissues were submerged in varying ethanol concentrations for another 30 min to rehydrate them and soaked for 10 min in distilled water. The cell nuclei were stained by being soaked in hematoxylin for 30 s and were washed with distilled water for several minutes afterward. The cell nuclei were then stained using eosin for two to five minutes and rewashed using distilled water. The cells were dried by immersing them in different ethanol concentrations for 30 s following the staining procedure. Xylene was utilized for the cleaning procedures after two rounds of dehydration were completed. Finally, the tissues were sealed with a cover slip.

### 4.8. Statistical Analysis

The one-way analysis of variance or ANOVA was performed through the use of a digital statistical software called the Statistical Package for the Social Sciences or SPSS Statistics 22.0 computer, and data were expressed as mean ± SD. When the F value is deemed notable, Duncan’s test is used to differentiate the comparisons between the groups, and resulting in a *p* < 0.05 would be a significant difference.

## 5. Conclusions

This study focused on the most active component of sesame that can lower blood glucose levels and its mechanism of action in regulating blood glucose. In this animal experiment, it was found that the rats treated with sesamin could lower blood sugar and reduce HbA1C. In histopathological examination, it was discovered that rats treated with sesamin had more pancreatic B cells, which stimulated insulin secretion. In addition, sesamin’s capacity to inhibit key processes in lipid synthesis and enhance cholesterol metabolism revealed its potential to ameliorate the adverse consequences of diabetes. Observations of the livers of diabetic rats treated with sesamin revealed a considerable decrease in the activity of AST and ALT enzymes, which may imply a reversal of the adverse effects of high blood glucose in diabetic rats and amelioration of liver damage caused by STZ. As sesame seeds are frequently added to breakfast or other meals, this study encourages their potential role in alleviating the symptoms of diabetes under a certain dose of dietary intake.

## Figures and Tables

**Table 1:** Effects of sesamin on serum biochemical amounts in STZ-induced diabetic rats.

Group ^1^	ALT ^2^ (U/L)	AST (U/L)	TG (mg/dL)	TC (mg/dL)	HDL (mg/dL)	Amylase (U/L)
Control	39 ± 5 ^b2^	126 ± 15 ^b^	75 ± 10.3 ^b^	72 ± 5.1 ^b^	21 ± 1.7 ^a^	1866 ± 129 ^a^
STZ	82 ± 11 ^a^	193 ± 32 ^a^	103 ± 9.8 ^a^	70 ± 2.9 ^b^	17 ± 2.5 ^b^	1423 ± 237 ^b^
STZ + SML	67 ± 12 ^b^	153 ± 27 ^b^	80 ± 8.2 ^b^	72 ± 5.3 ^b^	18 ± 2.2 ^ab^	1965 ± 391 ^a^
STZ + SMH	58 ± 14 ^b^	143 ± 24 ^b^	50 ± 6.7 ^c^	70 ± 2.6 ^b^	21 ± 2.3 ^a^	2124 ± 286 ^a^
STZ + MFM	57 ± 13 ^b^	122 ± 18 ^b^	68 ± 3.3 ^b^	80 ± 2.4 ^a^	21 ± 2.1 ^a^	1809 ± 154 ^a^

^1^ Control: streptozotocin (STZ: 55 mg/kg/body weight, intraperitoneal injection), sesamin low dose (SML: 20 mg/kg weight body/day), sesamin high dose (SMH: 60 mg/kg weight body/day), positive control: metformin (MFM: 250 mg/kg weight body/day); and ^2^ mean ± SD (*n* = 5), *p* < 0.05. Abbreviations: alanine aminotransferases, ALT; aspartate aminotransferase, AST; triglyceride, TG; total cholesterol, TC; and high-density lipoprotein, HDL. Values not sharing a common lower case letter are significantly different (*p* < 0.05).

**Table 2:** Experimental design.

No.	Group	Dose of Sample (mg/kg Body Weight/Day)	STZ (55 mg/kg Body Weight)	Animal Number
1	Control	-	**-**	6
2	Streptozotocin (STZ)	-	**+**	6
3	Sesamin low dose (SML)	20	**+**	6
4	Sesamin high dose (SMH)	60	**+**	6
5	Metformin (MFM)	250	**+**	6

## Data Availability

The data files generated and presented in this study are available upon request of the corresponding author.

## Sample Availability

Samples of the sesamin is available from the authors.

## References

[1] Diabetes. https://www.who.int/news-room/fact-sheets/detail/diabetes.

[2] Gouni-Berthold I., Krone W. (2006). Diabetic Ketoacidosis and Hyperosmolar Hyperglycemic State. Med. Klin..

[3] Whiting D.R., Guariguata L., Weil C., Shaw J. (2011). IDF Diabetes Atlas: Global Estimates of the Prevalence of Diabetes for 2011 and 2030. Diabetes Res. Clin. Pract..

[4] Bell A., Sherrell Z. (2022). How Do Diabetes Rates Vary by Country?. https://www.medicalnewstoday.com/articles/diabetes-rates-by-country.

[5] Li H., Chang G.-Y., Jiang Y.-H., Xu L., Shen L., Gu Z.-C., Lin H.-W., Shi F.-H. (2022). System Dynamic Model Simulates the Growth Trend of Diabetes Mellitus in Chinese Population: Implications for Future Urban Public Health Governance. Int. J. Public Health.

[6] Namiki M. (2007). Nutraceutical Functions of Sesame: A Review. Crit. Rev. Food Sci. Nutr..

[7] Mahendra Kumar C., Singh S.A. (2015). Bioactive Lignans from Sesame (*Sesamum indicum* L.): Evaluation of Their Antioxidant and Antibacterial Effects for Food Applications. J. Food Sci. Technol..

[8] Andargie M., Vinas M., Rathgeb A., Möller E., Karlovsky P. (2021). Lignans of Sesame (*Sesamum indicum* L.): A Comprehensive Review. Molecules.

[9] Kanu P., Bahsoon J., Kanu J., Kandeh J. (2010). Nutraceutical Importance of Sesame Seed and Oil: A Review of the Contribution of Their Lignans. Sierra Leone J. Biomed. Res..

[10] Wei P., Zhao F., Wang Z., Wang Q., Chai X., Hou G., Meng Q. (2022). Sesame (*Sesamum indicum* L.): A Comprehensive Review of Nutritional Value, Phytochemical Composition, Health Benefits, Development of Food, and Industrial Applications. Nutrients.

[11] Pathak N., Rai A.K., Kumari R., Bhat K.V. (2014). Value Addition in Sesame: A Perspective on Bioactive Components for Enhancing Utility and Profitability. Pharmacogn. Rev..

[12] Khatkar A., Sharma K.K. (2020). Phenylpropanoids and Its Derivatives: Biological Activities and Its Role in Food, Pharmaceutical and Cosmetic Industries. Crit. Rev. Food Sci. Nutr..

[13] Ciarcià G., Bianchi S., Tomasello B., Acquaviva R., Malfa G.A., Naletova I., La Mantia A., Di Giacomo C. (2022). Vitamin E and Non-Communicable Diseases: A Review. Biomedicines.

[14] Ma X., Wang Z., Zheng C., Liu C. (2022). A Comprehensive Review of Bioactive Compounds and Processing Technology of Sesame Seed. Oil Crop Sci..

[15] Orsavova J., Misurcova L., Ambrozova J., Vicha R., Mlcek J. (2015). Fatty Acids Composition of Vegetable Oils and Its Contribution to Dietary Energy Intake and Dependence of Cardiovascular Mortality on Dietary Intake of Fatty Acids. Int. J. Mol. Sci..

[16] Hirata F., Fujita K., Ishikura Y., Hosoda K., Ishikawa T., Nakamura H. (1996). Hypocholesterolemic Effect of Sesame Lignan in Humans. Atherosclerosis.

[17] Phitak T., Pothacharoen P., Settakorn J., Poompimol W., Caterson B., Kongtawelert P. (2012). Chondroprotective and Anti-Inflammatory Effects of Sesamin. Phytochemistry.

[18] Sohel M., Islam M.N., Hossain M.A., Sultana T., Dutta A., Rahman M.S., Aktar S., Islam K., Al Mamun A. (2022). Pharmacological Properties to Pharmacological Insight of Sesamin in Breast Cancer Treatment: A Literature-Based Review Study. Int. J. Breast Cancer.

[19] Peñalvo J.L., Hopia A., Adlercreutz H. (2006). Effect of Sesamin on Serum Cholesterol and Triglycerides Levels in LDL Receptor-Deficient Mice. Eur. J. Nutr..

[20] Majdalawieh A.F., Massri M., Nasrallah G.K. (2017). A Comprehensive Review on the Anti-Cancer Properties and Mechanisms of Action of Sesamin, a Lignan in Sesame Seeds (*Sesamum indicum*). Eur. J. Pharmacol..

[21] Xu P., Cai F., Liu X., Guo L. (2015). Sesamin Inhibits Lipopolysaccharide-Induced Proliferation and Invasion through the P38-MAPK and NF-ΚB Signaling Pathways in Prostate Cancer Cells. Oncol. Rep..

[22] Rosalina R., Weerapreeyakul N. (2021). An Insight into Sesamolin: Physicochemical Properties, Pharmacological Activities, and Future Research Prospects. Molecules.

[23] Nagarajan S., Lee J.K. (2021). Therapeutic Effects of Sesamolin on Leukemia Induced by WEHI-3B in Model Mice. Appl. Biol. Chem..

[24] Li Q.-Z., Zuo Z.-W., Liu Y. (2022). Recent Status of Sesaminol and Its Glucosides: Synthesis, Metabolism, and Biological Activities. Crit. Rev. Food Sci. Nutr..

[25] Grougnet R., Magiatis P., Laborie H., Lazarou D., Papadopoulos A., Skaltsounis A.-L. (2012). Sesamolinol Glucoside, Disaminyl Ether, and Other Lignans from Sesame Seeds. J. Agric. Food Chem..

[26] Majdalawieh A.F., Mansour Z.R. (2019). Sesamol, a Major Lignan in Sesame Seeds (*Sesamum indicum*): Anti-Cancer Properties and Mechanisms of Action. Eur. J. Pharmacol..

[27] Hong L., Yi W., Liangliang C., Juncheng H., Qin W., Xiaoxiang Z. (2013). Hypoglycaemic and Hypolipidaemic Activities of Sesamin from Sesame Meal and Its Ability to Ameliorate Insulin Resistance in KK-Ay Mice. J. Sci. Food Agric..

[28] Singh R.B., Mengi S.A., Xu Y.-J., Arneja A.S., Dhalla N.S. (2002). Pathogenesis of Atherosclerosis: A Multifactorial Process. Exp. Clin. Cardiol..

[29] Ahmad S., ElSherbiny N.M., Jamal M.S., Alzahrani F.A., Haque R., Khan R., Zaidi S.K., AlQahtani M.H., Liou G.I., Bhatia K. (2016). Anti-Inflammatory Role of Sesamin in STZ Induced Mice Model of Diabetic Retinopathy. J. Neuroimmunol..

[30] Kong P., Chen G., Jiang A., Wang Y., Song C., Zhuang J., Xi C., Wang G., Ji Y., Yan J. (2016). Sesamin Inhibits IL-1β-Stimulated Inflammatory Response in Human Osteoarthritis Chondrocytes by Activating Nrf2 Signaling Pathway. Oncotarget.

[31] Yan L.-J. (2022). The Nicotinamide/Streptozotocin Rodent Model of Type 2 Diabetes: Renal Pathophysiology and Redox Imbalance Features. Biomolecules.

[32] Li X., Gao Y., Li S., Yang J. (2015). Effect of Sesamin on Pulmonary Vascular Remodeling in Rats with Monocrotaline-Induced Pulmonary Hypertension. Zhongguo Zhong Yao Za Zhi.

[33] Noguchi T., Ikeda K., Sasaki Y., Yamamoto J., Yamori Y. (2004). Effects of vitamin E and sesamin on hypertension and cerebral thrombogenesis in stroke-prone spontaneously hypertensive rats. Clin. Exp. Pharmacol. Physiol..

[34] Nakano D., Kwak C.-J., Fujii K., Ikemura K., Satake A., Ohkita M., Takaoka M., Ono Y., Nakai M., Tomimori N. (2006). Sesamin Metabolites Induce an Endothelial Nitric Oxide-Dependent Vasorelaxation through Their Antioxidative Property-Independent Mechanisms: Possible Involvement of the Metabolites in the Antihypertensive Effect of Sesamin. J. Pharmacol. Exp. Ther..

[35] Thuy T.D., Phan N.N., Wang C.-Y., Yu H.-G., Wang S.-Y., Huang P.-L., Do Y.-Y., Lin Y.-C. (2017). Novel Therapeutic Effects of Sesamin on Diabetes-Induced Cardiac Dysfunction. Mol. Med. Rep..

[36] Ananda P.K., Kumarappan C., Christudas S., Kalaichelvan V. (2012). Effect of Biophytum Sensitivum on Streptozotocin and Nicotinamide-Induced Diabetic Rats. Asian Pac. J. Trop. Biomed..

[37] Ramesh B., Saravanan R., Pugalendi K.V. (2005). Influence of Sesame Oil on Blood Glucose, Lipid Peroxidation, and Antioxidant Status in Streptozotocin Diabetic Rats. J. Med. Food.

[38] Yargholi A., Najafi M.H., Zareian M.A., Hawkins J., Shirbeigi L., Ayati M.H. (2021). The Effects of Sesame Consumption on Glycemic Control in Adults: A Systematic Review and Meta-Analysis of Randomized Clinical Trial. Evid. -Based Complement. Altern. Med..

[39] Bigoniya P., Nishad R., Singh C.S. (2012). Preventive Effect of Sesame Seed Cake on Hyperglycemia and Obesity against High Fructose-Diet Induced Type 2 Diabetes in Rats. Food Chem..

[40] Lei H., Han J., Wang Q., Guo S., Sun H., Zhang X. (2012). Effects of Sesamin on Streptozotocin (STZ)-Induced NIT-1 Pancreatic β-Cell Damage. Int. J. Mol. Sci..

[41] Eleazu C.O., Eleazu K.C., Chukwuma S., Essien U.N. (2013). Review of the Mechanism of Cell Death Resulting from Streptozotocin Challenge in Experimental Animals, Its Practical Use and Potential Risk to Humans. J. Diabetes Metab. Disord..

[42] Cerf M.E. (2013). Beta Cell Dysfunction and Insulin Resistance. Front. Endocrinol..

[43] Dworzański J., Strycharz-Dudziak M., Kliszczewska E., Kiełczykowska M., Dworzańska A., Drop B., Polz-Dacewicz M. (2020). Glutathione Peroxidase (GPx) and Superoxide Dismutase (SOD) Activity in Patients with Diabetes Mellitus Type 2 Infected with Epstein-Barr Virus. PLoS ONE.

[44] Madi M., Babu S., Kumari S., Shetty S., Achalli S., Madiyal A., Bhat M. (2016). Status of Serum and Salivary Levels of Superoxide Dismutase in Type 2 Diabetes Mellitus with Oral Manifestations: A Case Control Study. Ethiop. J. Health Sci..

[45] Singhai M., Goyal R., Faizy A. (2011). Glutathione Peroxidase Activity in Obese and Nonobese Diabetic Patients and Role of Hyperglycemia in Oxidative Stress. J. Midlife Health.

[46] Farbood Y., Ghaderi S., Rashno M., Khoshnam S.E., Khorsandi L., Sarkaki A., Rashno M. (2019). Sesamin: A Promising Protective Agent against Diabetes-Associated Cognitive Decline in Rats. Life Sci..

[47] Kong X., Wang G.-D., Ma M.-Z., Deng R.-Y., Guo L.-Q., Zhang J.-X., Yang J.-R., Su Q. (2015). Sesamin Ameliorates Advanced Glycation End Products-Induced Pancreatic β-Cell Dysfunction and Apoptosis. Nutrients.

[48] Sharma T., Singh I., Singh N., Vardey G., Kumawat M., Ghalaut V., Shankar V. (2013). Antioxidant Enzymes and Lipid Peroxidation in Type 2 Diabetes Mellitus Patients with and without Nephropathy. N. Am. J. Med. Sci..

[49] Lubos E., Loscalzo J., Handy D.E. (2011). Glutathione Peroxidase-1 in Health and Disease: From Molecular Mechanisms to Therapeutic Opportunities. Antioxid. Redox Signal.

[50] Hirano T. (2018). Pathophysiology of Diabetic Dyslipidemia. J. Atheroscler. Thromb..

[51] Lemieux I., Lamarche B., Couillard C., Pascot A., Cantin B., Bergeron J., Dagenais G.R., Després J.-P. (2001). Total Cholesterol/HDL Cholesterol Ratio vs LDL Cholesterol/HDL Cholesterol Ratio as Indices of Ischemic Heart Disease Risk in Men. Arch. Intern. Med..

[52] Kwon Y.H., Kim S.-K., Cho J.H., Kwon H., Park S.E., Oh H.-G., Park C.-Y., Lee W.-Y., Oh K.-W., Park S.-W. (2018). The Association between Persistent Hypertriglyceridemia and the Risk of Diabetes Development: The Kangbuk Samsung Health Study. Endocrinol. Metab..

[53] Yadav R., Bhartiya J.P., Verma S.K., Nandkeoliar M.K. (2013). The Evaluation of Serum Amylase in the Patients of Type 2 Diabetes Mellitus, with a Possible Correlation with the Pancreatic Functions. J. Clin. Diagn. Res..

[54] Akanya H., Isa U., Adeyemi H., Ossamulu I. (2015). Effect of *Sesamum indicum* (Linn) Seeds Supplemented Diets on Blood Glucose, Lipid Profiles and Serum Levels of Enzymes in Alloxan Induced Diabetic Rats. J. Appl. Life Sci. Int..

[55] Mohammad Shahi M., Zakerzadeh M., Zakerkish M., Zarei M., Saki A. (2017). Effect of Sesamin Supplementation on Glycemic Status, Inflammatory Markers, and Adiponectin Levels in Patients with Type 2 Diabetes Mellitus. J. Diet. Suppl..

[56] Tai T.-S., Tien N., Shen H.-Y., Chu F.-Y., Wang C.C.N., Lu C.-H., Yu H.-I., Kung F.-P., Chuang H.-H., Lee Y.-R. (2019). Sesamin, a Naturally Occurring Lignan, Inhibits Ligand-Induced Lipogenesis through Interaction with Liver X Receptor Alpha (LXR *α*) and Pregnane X Receptor (PXR). Evid.-Based Complement. Altern. Med..

[57] Majdalawieh A.F., Dalibalta S., Yousef S.M. (2020). Effects of Sesamin on Fatty Acid and Cholesterol Metabolism, Macrophage Cholesterol Homeostasis and Serum Lipid Profile: A Comprehensive Review. Eur. J. Pharmacol..

[58] Jeon C.Y., Roberts C.K., Crespi C.M., Zhang Z.-F. (2013). Elevated Liver Enzymes in Individuals with Undiagnosed Diabetes in the U.S. J. Diabetes Complicat..

[59] Shrestha N., Bhatt N., Neopane P., Dahal S., Regmi P., Khanal M., Shrestha R. (2017). Hepatic Involvement with Elevated Liver Enzymes in Nepalese Subjects with Type 2 Diabetes Mellitus. Int. J. Biochem. Res. Rev..

[60] Ohaeri O.C. (2001). Effect of Garlic Oil on the Levels of Various Enzymes in the Serum and Tissue of Streptozotocin Diabetic Rats. Biosci. Rep..

